# Methyltransferase-like 3 induces the development of cervical cancer by enhancing insulin-like growth factor 2 mRNA-binding proteins 3-mediated apoptotic chromatin condensation inducer 1 mRNA stability

**DOI:** 10.1080/21655979.2022.2044261

**Published:** 2022-03-08

**Authors:** Cuihong Su, Yan Zhang, Ping Chen, Wei Yang, Jiaqiu Du, Danfeng Zhang

**Affiliations:** Department of Obstetrics and Gynecology, First Affiliated Hospital of Jiamusi University, Jiamusi, Heilongjiang, China

**Keywords:** Cervical cancer, N6-methyladenosine, METTL3, ACIN1, IGF2BP3

## Abstract

N6-methyladenosine (m6A) plays a critical role in the tumorigenesis of cervical cancer (CC). Here, we aimed to investigate the potential role of methyltransferase-like 3 (METTL3) in CC. Gene expression was determined via real-time quantitative polymerase chain reaction. Cellular functions were detected using colony formation, 5-ethynyl-2′-deoxyuridine (EdU), and Transwell assays. The interactions among METTL3, insulin-like growth factor 2 mRNA-binding protein 3 (IGF2BP3), and apoptotic chromatin condensation inducer 1 (ACIN1) were confirmed using the MeRIP and RIP assays. An *in vivo* assay was performed to verify the role of METTL3 in CC development. METTL3 is overexpressed in CC, and therefore, its knockdown inhibits the proliferation and migration of CC cells. Silencing METTL3 inhibits tumor growth *in vivo*. Moreover, a positive association was observed between METTL3 and ACIN1. METTL3 interacts with IGF2BP3 to promote the mRNA stability of ACIN1, the overexpression of which induces the aggressiveness of CC cells. METTL3 promotes ACIN1 mRNA stability to accelerate CC progression, implying that METTL3 is a promising biomarker in CC.

## Graphical abstract

**Figure:**
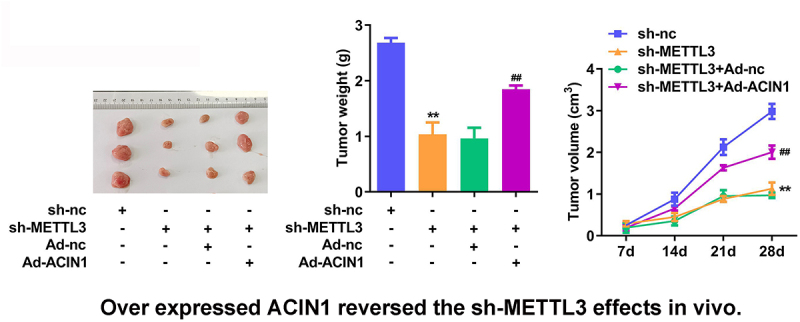

## Introduction

Cervical cancer (CC) is ranked fourth among the most prevalent gynecological tumors and remains one of the leading causes of cancer-related deaths worldwide [1,2]. Approximately 570,000 new cases and over 300,000 mortalities are registered annually [3]. In addition, CC poses a severe challenge to healthcare worldwide. It is widely acknowledged that the pathogenesis of CC is a complicated process involving multiple factors, such as human papillomavirus (HPV) infection, smoking, sexual hygiene, and genetic alternations [4–6]. Notably, HPV infection has enormous significance in the development of CC [7]. Owing to HPV vaccination and improved early diagnostic screening, the prevention, and treatment of CC have made great strides. Nonetheless, the long-term prognosis of CC patients remains poor [8]. Accordingly, it is essential to elucidate the underlying mechanism of CC and identify effective biomarkers of CC progression.

Various lines of evidence have demonstrated that epigenetic modifications play a core role in the occurrence and evolution of human cancers [9,10]. Epigenetic modifications trigger reversible changes in nucleic acids and related proteins without altering the DNA base sequence [11,12]. With the breakthrough discovery of epigenetics, post-transcriptional RNA modification has become a hotspot in cancer research [13]. According to the MODOMICS database, there are more than 100 RNA modifications at the post-transcriptional level [14]. Moreover, N6‐methyladenosine (m6A) modification has been identified as the most dominant RNA modification in eukaryotes [15]. Numerous studies have shown that m6A plays a pivotal role in the tumorigenicity of diverse malignancies, including CC [16–19]. For instance, attenuation of m6A modification promotes malignant traits and the activation of the Wnt/ phosphatidylinositol-3-kinase (PI3K)- protein kinase B (Akt) pathway in gastric cancer [20]. The long non-coding RNA (lncRNA) zinc finger antisense 1 (ZFAS1) accelerates CC development by sequestering miR-647 via m6A modification [21]. Nevertheless, further mechanistic studies are needed to gain a comprehensive understanding of m6A modification in CC.

Methyltransferase-like 3 (METTL3), a crucial m6A regulatory enzyme, governs the process of m6A methylation, thereby regulating the tumor formation and development by affecting mRNA stability, translation, or degradation [22,23]. In recent years, the potential of METTL3 as a tumor biomarker has been ascertained to correlate with the malignancy of cancer cells [24]. Accumulating evidence suggests that METTL3 exhibits tumorigenic functions in a wide range of malignancies, including breast [25], colorectal [26], and gastric cancers [27]. More importantly, METTL3 contributes to the aggressive features of CC cells [28]. Unfortunately, its mode of action in CC has not been fully elucidated.

Hence, the current study aimed to explore of the latent molecular mechanism of METTL3 in the progression of CC. We hypothesized that silencing of METTL3 retarded CC cell growth and migration by weakening apoptotic chromatin condensation inducer 1 (ACIN1) mRNA stability via an m6A-insulin-like growth factor 2 mRNA-binding proteins 3 (IGF2BP3)-dependent mechanism.

## Material and methods

### Tissue samples

The present research passed the approval of the Ethics Committee of First Affiliated Hospital of Jiamusi University (2020–103-02). This study was carried out by collecting tumor samples and non-tumor tissues of CC patients from First Affiliated Hospital of Jiamusi University. In brief, the non-tumor tissues that did not contain obvious cancer cells were collected 5–10 cm away from the border of the tumor. Subsequently, the tumor and the para-carcinoma tissues were fixed in 10% formalin. No antitumor therapy (chemotherapy or immunotherapy)was conducted for participants before cervicectomy. All patients signed the informed consent prior to inclusion. Tissue samples were preserved at -80°C until utilization.

### Real-time quantitative polymerase chain reaction

According to a previous study [29], the TRIzol kit (Invitrogen) was applied for total RNA isolation according to the product directions. The RNA concentration was analyzed using NanoDrop 2000 (Thermo, America). The cDNAs were synthesized by using the PrimeScript RT reagent kit transcriptase, Random 6mers, RNase inhibitor, Oligo dT primer, a dNTP mixture, and reaction buffer. The expression of mRNAs was analyzed by RT-qPCR in ABI7300 PCR apparatus (Applied Biosystems, Carlsbad, USA) by means of SYBR-Green Master Mix Kit (Takara, Tokyo, Japan). The cycle conditions used were: 95°C for 30s, followed by 95°C for 5 s and 40 cycles at 60°C for 30s each.The relative expression levels of genes were normalized to β-actin via 2-^ΔΔCt^ method. The primers used were as follows: METTL3 (forward: 5′-TTGTCTCCAACCTTCCGTAG-3′; reverse: 5′-CCAGATCAGAGAGGTGGTGTAG-3′); ACIN1 (forward: 5′-AGGTTAGGCAAGGAGGTGGT-3′; reverse: 5′-TGTTCCCAAGAGAAGGCTGT-3′); IGF2BP3 (forward: 5′-AGTTGTTGTCCCTCGTGACC′; reverse: 5′-GTCCACTTTGCAGAGCCTTC-3′), and β-actin (forward: 5′-CTCCATCCTGGCCTCGCTGT-3′; reverse: 5′-GCTGCTACCTTACCGTTCC-3′).

### Cell culture

The human normal cervical epithelial cell line (End1) and CC cell lines (HeLa and SiHa) were provided by ATCC (Manassas, USA) and maintained in media consisting of DMEM (Gibco™, Waltham, USA), 10% FBS (Invitrogen, Carlsbad, USA), and 1% penicillin/streptomycin (Invitrogen). Cells were cultured in a humid atmosphere with 5% CO_2_ at 37°C.

### Cell transfection

According to a previous study [30], specific small interfering RNAs (siRNAs) for METTL3 were constructed by Genepharma (Shanghai, China), with nonspecific siRNAs as a negative control. To upregulate insulin-like growth factor 2 mRNA-binding protein 3 (IGF2BP3) and apoptotic chromatin condensation inducer 1 (ACIN1), their cDNA fragments were amplified and inserted into pcDNA3.1 vectors to generate IGF2BP3-overexpressing (OE-IGF2BP3) and ACIN1-overexpressing (OE- ACIN1) plasmids. These vectors were transfected into HeLa and SiHa cells using the Lipofectamine 3000 kit (Invitrogen), according to the manufacturer’s protocols. For stable knockdown of METTL3, HeLa cells were infected with lentiviral vectors encoding a short hairpin RNA (shRNA) targeting METTL3, or scrambled shRNA (sh-NC) purchased from GeneChem (Shanghai, China), using 5 μg/mL polybrene. Stably infected cells were screened using 4 μg/mL puromycin treatment for two weeks.

### Cell proliferation assays

According to a previous study [31], for the colony formation assay, transfected HeLa and SiHa cells were inoculated into 6-well culture plates at a density of 500 cells/well and incubated under the indicated conditions for two weeks. After fixation in 4% paraformaldehyde, CC cells were stained with 0.1% crystal violet (Sigma-Aldrich). For the 5-ethynyl-2′-deoxyuridine (EdU) assay, the collected CC cells were treated with an EdU staining kit (Ribobio, Guangzhou, China) according to the manufacturer’s instructions. DAPI was used for nuclear staining, and images were captured using a fluorescence microscope (Leica, Wetzlar, Germany).

### Cell migration assay

According to a previous study [32], transwell assays were performed to estimate the migration of CC cells using a Transwell system (Corning, New York, USA). HeLa and SiHa cells were digested with trypsin, collected, and placed in the upper chamber containing a 200-μL serum-free medium. The bottom of the Transwell chamber was supplemented with 600 μL DMEM supplemented with 20% FBS. After 24 h of incubation, the CC cells that passed through the filter were immobilized with 4% paraformaldehyde, stained with 0.1% crystal violet, and observed under a microscope.

### Western blot analysis

According to a previous study [33], HeLa and SiHa cells were lysed in RIPA lysis buffer (Thermo Fisher Scientific, Waltham, USA) with a protease inhibitor, and a BCA assay kit was used to determine protein concentration (Beyotime, Shanghai, China). Protein extracts were loaded on 10% SDS-PAGE gels, transferred onto PVDF membranes, blocked in 5% skimmed milk, and probed with the corresponding primary antibodies at 4°C overnight. Subsequently, the membranes were treated with secondary antibodies for 2 h at room temperature and measured using an enhanced chemiluminescence (ECL) kit (Thermo Fisher Scientific). The following primary antibodies were used: anti-METTL3 (Abcam, Cambridge, UK), anti-IGF2BP3 (Millipore, Billerica, USA), anti-ACIN1, and anti-GAPDH (Cell Signaling Technology, St. Louis, USA). GAPDH served as an internal control.

### RNA stability assay

According to a previous study [34], Actinomycin D (Act-D; MedChemExpress, Beijing, China) was used to examine RNA stability. Briefly, HeLa and SiHa cells were treated with 5 μg/ml Act-D. At 0, 1, 2, 4, and 8 h post-incubation, RT-qPCR was conducted to detect the mRNA expression of ACIN1.

### Cycloheximide chase assay

According to a previous study [35], to measure protein stability, CC cells were treated with 50 μg/ml cycloheximide (Sigma) and cultured for 0, 1, 2, 4, and 8 h. The cells were then subjected to Western blot analysis to detect ACIN1 protein expression levels.

**In vivo** *assay*

All experimental procedures were carried out under the approval of the Institutional Animal Care and Use Committee of the Institutional Animal Care and Utilization Committee of First Affiliated Hospital of Jiamusi University. According to a previous study [36], approximately six-week-old BALB/c nude mice were used to establish xenograft models. Briefly, 2 × 10^6^ stably transfected HeLa cells were subcutaneously inoculated into the left flank of mice. Afterward, nude mice were reared for four weeks, and the size of the xenografts was monitored weekly using a Vernier caliper. Following euthanasia, the xenografts were excised from the mice and weighed.

### m6A-RNA immunoprecipitation (MeRIP)

According to a previous study [37], the MeRIP experiment was performed to identify the m6A modification of ACIN1, following the recommendations of the Magna MeRIP™ m6A kit (Millipore, MA, USA). Following RNA isolation, total RNA was fragmented into nucleotide debris and immunoprecipitated with an anti-m6A antibody or IgG using protein A/G magnetic beads (Pierce, Rockford, USA). Bound RNAs were eluted from magnetic beads, subjected to proteinase K digestion, and quantified via RT-qPCR.

### RIP assay

According to a previous study [38], the Magna RIP RNA-Binding Protein Immunoprecipitation Kit (Millipore) was utilized to perform the RIP assay according to the product manuals. Cell lysates were treated with magnetic beads and a specific antibody for IGF2BP3 (Millipore), with IgG (Millipore) as a negative control, at 4°C overnight. The enrichment of ACIN1 in the RNA precipitates was measured using RT-qPCR.

### Statistical analysis

All statistical results are expressed as the mean ± SD, and each experiment was performed in triplicate. Data processing and analysis were performed using SPSS (version 21.0; SPSS, Chicago, USA). The student’s t-test and one-way analysis of variance were used for statistical comparisons. The overall survival rate of CC patients was estimated using Kaplan-Meier analysis and log-rank test. Pearson’s correlation analysis was employed to assess the relationships between METTL3, ACIN1, and IGF2BP3. Differences were considered statistically significant at *p* < 0.05.

## Results

This study illustrated that elevated METTL3 levels were associated with poor prognosis in of CC patients. Notably, the silencing of METTL3 retarded CC cell growth and migration by weakening ACIN1 mRNA stability via an m6A-IGF2BP3-dependent mechanism, expanding our in-depth knowledge of METTL3 in CC progression and unraveling the possibility of using METTL3 as a therapeutic target for CC therapy.

### High expression of METTL3 predicted poor outcome in CC

**Figure 1.**
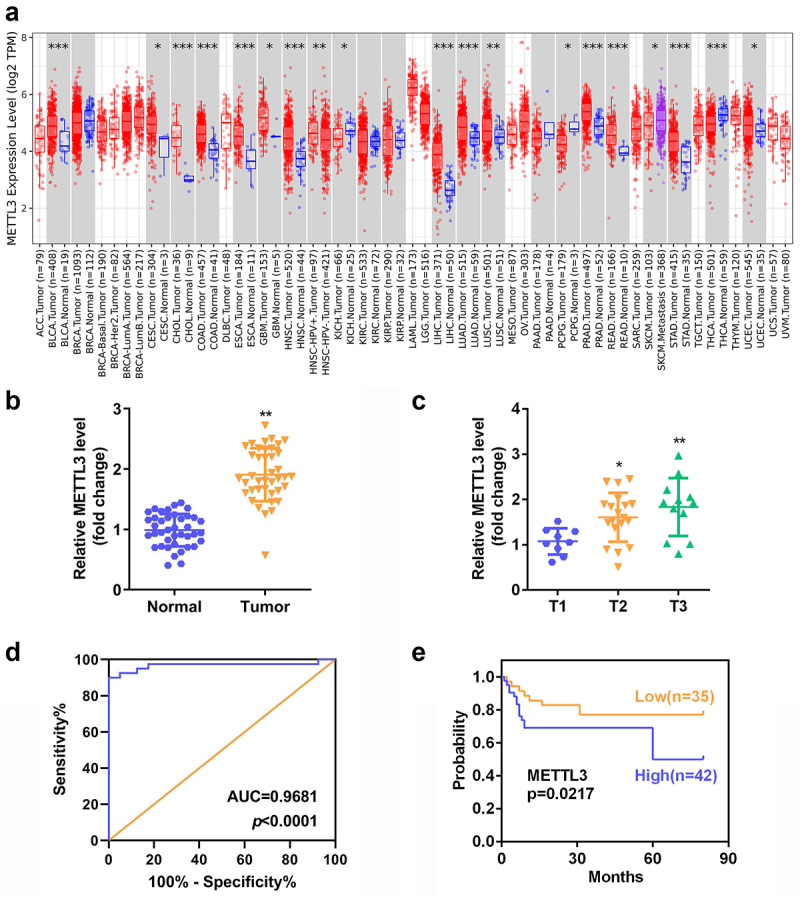
**High expression of METTL3 predicted poor outcome in CC**. (a) The expression profile of METTL3 in different malignant tumors. (b) METTL3 expression in CC tissues and normal samples. (c) RT-qPCR detection of METTL3 level in different clinical stages. (d) The AUC of METTL3 concerning CC patients. (e) The relationship between METTL3 expression and the prognosis of CC. *P < 0.05, **P < 0.01 and ***P < 0.001 vs normal or T1 group.

Using the gene expression profiling interactive analysis (GEPIA) database, METTL3 was found to be abnormally expressed in a wide range of malignant tumors, including CCs (Figure 1a). To verify the expression pattern of METTL3 in CC, we measured METTL3 levels via RT-qPCR analysis. CC tissues exhibited higher METTL3 expression than the normal tissues (Figure 1b). Clinically, the expression level of METTL3 gradually increased with the TNM stage (Figure 1c). Besides, METTL3 in CC patients achieved the highest AUC of 0.9681 (P < 0.0001) (Figure 1d). In addition, Kaplan-Meier analysis showed that high levels of METTL3 were associated with poor OS rates (Figure 1e).

### Knockdown of METTL3 suppressed CC cell proliferation and migration

**Figure 2.**
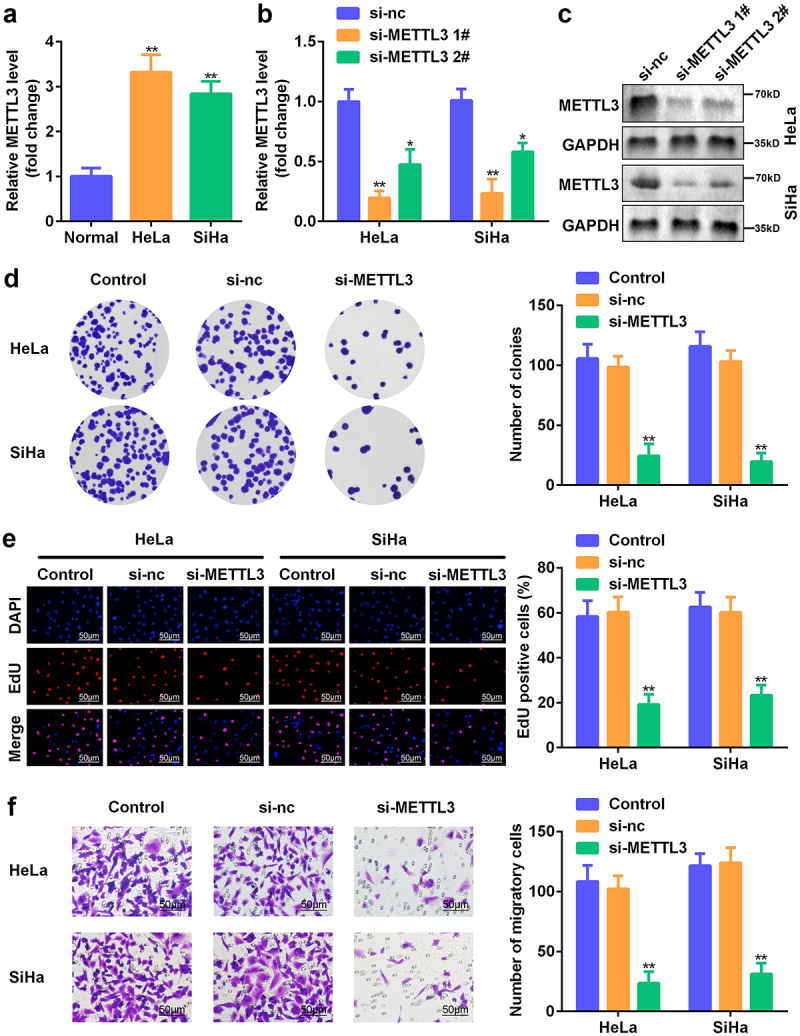
**Knockdown of METTL3 suppressed CC cell proliferation and migration**. (a) The expression of METTL3 in CC and normal cells. (b–c) Transfection efficiency was confirmed using RT-qPCR and Western blotting analysis. (d–e) The role of METTL3 in CC cell proliferation was assessed through colony formation and the EdU assay. (f) Transwell assay was performed to determine cell migration of HeLa and SiHa cells. *P < 0.05 and **P < 0.01 vs. si-NC group.

Next, we aimed to elucidate the potential role of METTL3 in CC development *in vitro*. As expected, METTL3 was abundantly expressed in CC cells compared to that in normal cervical epithelial cells (Figure 2a). Accordingly, METTL3 expression was downregulated in HeLa and SiHa cells in loss-of-function assays (Figure 2b). The Western blotting analysis further confirmed that the METTL3 protein level was remarkably reduced in HeLa and SiHa cells after transfection (Figure 2c). Our observations indicated that depletion of METTL3 diminished the number of colonies in CC cells (Figure 2d). The proliferative potency of METTL3 was confirmed using an EdU assay (Figure 2e). Moreover, the silencing of METTL3 led to the suppression of cell migration in HeLa and SiHa cells (figure 2f). Thus, METTL3 is a tumor promoter during CC progression.

### ACIN1 was positively modulated by METTL3

**Figure 3.**
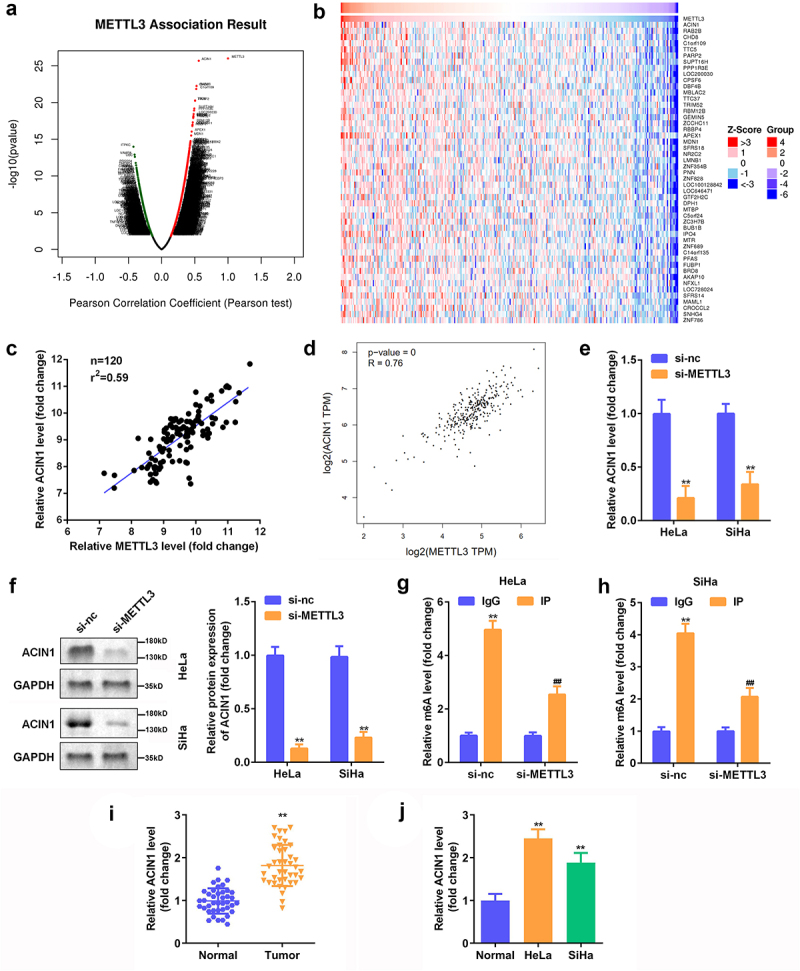
**ACIN1 was positively modulated by METTL3**. (a) The Pearson correlation coefficient between METTL3 and human genes in CC was analyzed with LinkedOmics. (b) The genes related to METTL3 in CC tissues were shown. (c) The correlation of METTL3 and ACIN1 in clinical samples. (d) The correlation of METTL3 and ACIN1 in CC tissues was analyzed using GEPIA. (e–f) RT-qPCR and Western blot analyses of ACIN1 expression in CC cells transfected with si-NC or si-METTL3. (g–h) MeRIP analysis of ACIN1 mRNA in CC cells transfected with si-NC or si-METTL3. (i-j) RT-qPCR of ACIN1 expression in CC tissues and cells. **P < 0.01 vs. si-NC group; **P < 0.01 vs. si-NC group.

Based on the above findings, we aimed to shed light on the molecular mechanism of METTL3 in CC pathogenesis. Using LinkedOmics, candidate genes associated with METTL3 in CC were identified (Figure 3a). ACIN1 was closely correlated with METTL3 among all the candidate genes related to METTL3 (Figure 3b). Pearson’s correlation analysis illustrated that METTL3 levels were positively correlated with the expression of ACIN1 in clinical samples collected from CC patients (Figure 3c). By browsing the GEPIA website, a positive correlation between METTL3 and ACIN1 was further confirmed (Figure 3d). In addition, the knockdown of METTL3 provoked a notable decrease in ACIN1 expression at both the mRNA and protein levels (Figure 3e–F). Moreover, the results of the MeRIP experiment justified that ACIN1 was enriched by m6A antibody in contrast to IgG, and silencing METTL3 significantly reduced the enrichment of ACIN1 in the m6A antibody, which suggested that the depletion of METTL3 caused the decreased m6A level of ACIN1 (Figure 3g–h). Additionally, RT-qPCR results showed that ACIN1 was increased in the CC tissues and cells (Figure 3i-j). Taken together, these results indicate that ACIN1 is a downstream effector of METTL3.

### METTL3 acted as an oncogene in CC by targeting ACIN1

**Figure 4.**
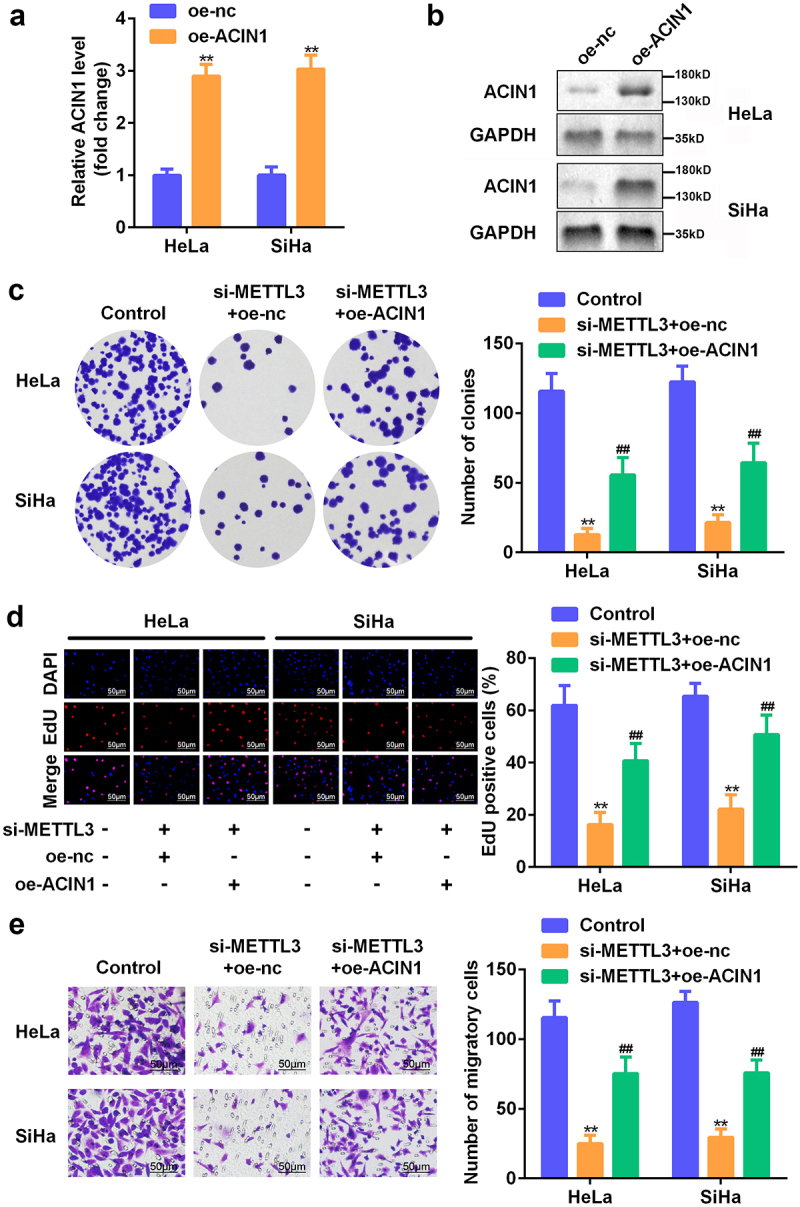
**METTL3 acted as an ACIN1-targeting oncogene in CC**. (a–b) Transfection efficiency was confirmed via RT-qPCR and Western blotting analysis. (c–d) The role of ACIN1 in CC cell proliferation was estimated through colony formation and the EdU assay. (e) Cell migratory ability was measured with the Transwell assay. **P < 0.01 vs. control group; ^##^*P* < 0.01 vs. si-METTL3+ OE-NC group.

Subsequently, we explored the involvement of ACIN1 on the effect of METTL3 on CC development. The transfection efficiency of ACIN1 overexpression was assessed using RT-qPCR (Figure 4a). Western blotting analysis validated that the protein expression of ACIN1 was enhanced after transfection (Figure 4b). The colony formation and EdU assays revealed that the impaired proliferative capacity of HeLa and SiHa cells triggered by METTL3 knockdown was abrogated when ACIN1 was overexpressed (Figure 4c–d). Consistently, our results indicated that the upregulation of ACIN1 counteracted the effects of METTL3 depletion on CC cell migration (Figure 4e). Overall, ACIN1 mediated the function of METTL3 in controlling the aggressive characteristics of CC cells.

### Over expressed ACIN1 reversed the sh-METTL3 effects on the CC cell growth in vivo

**Figure 5.**
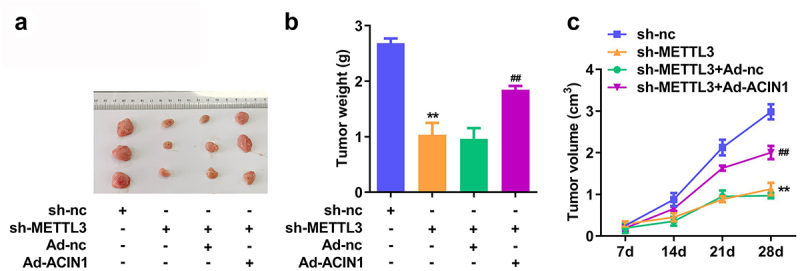
**Over expressed ACIN1 reversed the sh-METTL3 effects on the CC cell growth *in vivo.*** (a) Representative images of excised tumors. (b–c) The impacts of METTL3 knockdown and ACIN1 overexpression on tumor size and weight were analyzed. *P < 0.05 and **P < 0.01 vs. sh-nc group.

We investigated the impact of METTL3 and ACIN1 on tumor growth *in vivo*. To establish xenograft tumor models, HeLa cells stably transfected with sh-NC, sh-METTL3, Ad-nc and Ad-ACIN1 were injected into nude mice. As shown in Figure 5a, mice from the sh-METTL3 group presented smaller neoplasms than those from the sh-NC group. Ad-ACIN1 reversed the sh-METTL3 roles. The weight and growth curve of xenograft tumors showed that the METTL3 knockdown led to a significant reduction in the weight and volume of tumor tissues, while Ad-ACIN1 also reversed the sh-METTL3 roles (Figure 5b–c). Based on these data, we hypothesized that METTL3 downregulation would impede the growth of CC cells *in vivo*.

### METTL3 contributed to the mRNA stability of ACIN1

**Figure 6.**
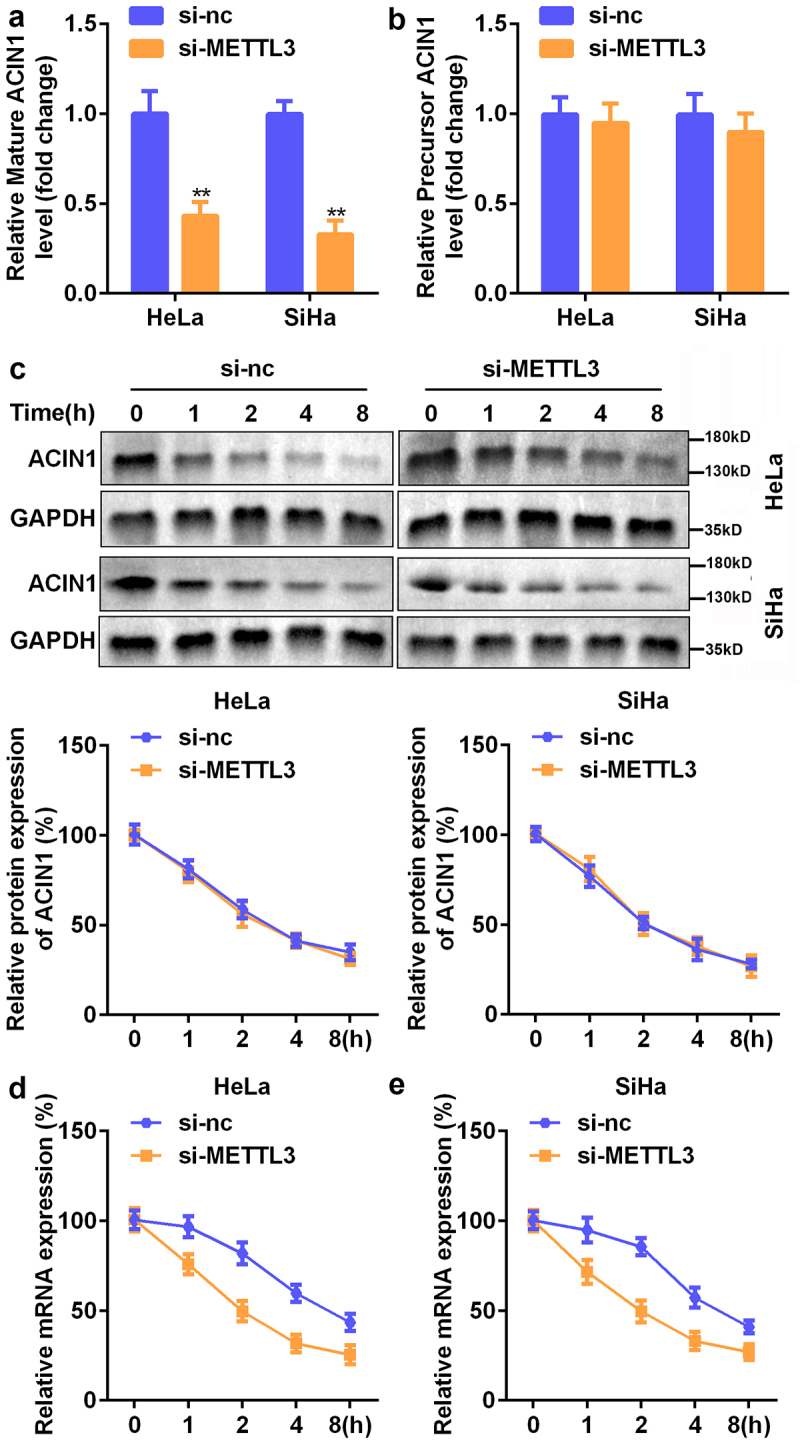
**METTL3 contributed to the mRNA stability of ACIN1**. (a–b) RT-qPCR analysis of mature and precursor mRNA expression of ACIN1. (c) Following the treatment of CC cells with cycloheximide, Western blot analysis was adopted to detect ACIN1 expression. (d) RT-qPCR analysis of the mRNA expression of ACIN1 in CC cells exposed to Act-D. **P < 0.01 vs. si-NC group.

Therefore, we further probed the mode of action of METTL3 in the modulation of ACIN1 expression. According to RT-qPCR analysis, silencing of METTL3 resulted in an evident decline in ACIN1 mRNA expression levels but failed to change the precursor ACIN1 level (Figure 6a–b). Likewise, the Western blotting analysis showed that the protein expression of ACIN1 in response to si-METTL3 was lower than that in response to si-NC (Figure 6c). Interestingly, we observed that the downregulation of METTL3 weakened the stability of ACIN1 mRNA in both HeLa and SiHa cells (Figure 6d–e).

### IGF2BP3 was responsible for the regulatory role of METTL3 in ACIN1 expression

**Figure 7.**
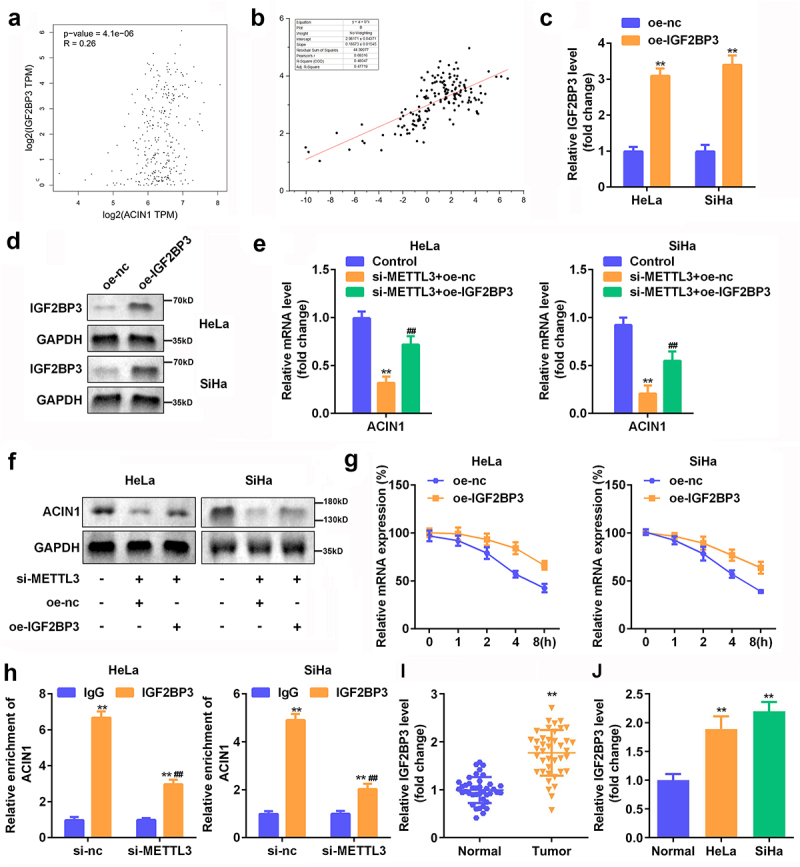
**IGF2BP3 was responsible for the regulatory role of METTL3 in ACIN1 expression**. (a) The correlation of IGF2BP3 and ACIN1 in CC was analyzed using GEPIA. (b) Pearson correlation analysis of the association between IGF2BP3 and ACIN1 in clinical tumor tissues from CC patients. (c–d) Transfection efficiency was confirmed by RT-qPCR and Western blot analysis. (e–f) The potential of METTL3/IGF2BP3 axis in ACIN1 expression was analyzed by RT-qPCR and Western blot analysis. (g) After Act-D treatment, the mRNA degradation of ACIN1 was examined via RT-qPCR. (h) RIP experiment was carried out to determine the influence of METTL3 downregulation on the abundance of ACIN1 in precipitates enriched by the IGF2BP3 antibody. (i-j) RT-qPCR of ACIN1 expression in CC tissues and cells. **P < 0.01 vs. OE-NC or IgG or Control group; ^##^*P* < 0.01 vs. si-METTL3+ OE-NC or si-NC group.

Since IGF2BP3 is a significant mediator of mRNA stability [39], we wondered whether it regulates ACIN1 by METTL3. Pearson’s correlation analysis of data from GEPIA revealed a positive association between ACIN1 expression and IGF2BP3 levels in CC samples (Figure 7a). Coincidentally, we verified that the expression level of ACIN1 was positively correlated with IGF2BP3 expression in clinical samples (Figure 7b). We overexpressed IGF2BP3 in HeLa and SiHa cells to identify the role of IGF2BP3 in METTL3-mediated ACIN1 expression. RT-qPCR and Western blotting analysis revealed that the expression of IGF2BP3 was upregulated following transfection (Figure 7c–d). Our observations demonstrated that an ectopic expression of IGF2BP3 reversed the decrease in ACIN1 levels caused by METTL3 knockdown (Figure 7e). The same trend at the protein level was validated using Western blotting analysis (figure 7f). More importantly, we confirmed that IGF2BP3 overexpression prolonged the half-life of ACIN1 mRNA in HeLa and SiHa cells (Figure 7g). Furthermore, the results of the RIP experiment justified that the expression of ACIN1 in complexes enriched with IGF2BP3 was overtly attenuated by the silencing of METTL3 (Figure 7h). Additionally, RT-qPCR results showed that ACIN1 was increased in the CC tissues and cells (Figure 7i-j). Overall, METTL3 enhanced the mRNA stability of ACIN1 in an IGF2BP3-dependent manner.

## Discussion

Based on its highest morbidity and mortality among malignancies of the female reproductive system, CC is a dominant cause of cancer-related deaths among women worldwide [40,41]. CC accounts for nearly 7.5% of women dying from cancer, representing a global health problem [42]. Therefore, this study aimed to gain insights into the etiology and mechanism of CC. Herein, we demonstrated that METTL3 facilitates tumor formation and CC progression. Mechanistically, METTL3 contributes to CC growth and migration by regulating the stabilization of ACIN1 mediated by IGF2BP3.

Currently, our understanding of m6A has dramatically expanded due to the in-depth study of epigenetic modifications. As the most abundant RNA modification, m6A has been shown to function as a critical participant in developing diverse diseases, including human cancer [43–46]. Furthermore, abnormal m6A modification is strongly related to tumor development and is predictive for cancer outcome [47–50]. Many studies have validated that METTL3 is a core modulator of m6A modification and acts as a promoting factor in the malignancy of multiple tumors, including CC. For example, METTL3 facilitates cell proliferation and metastasis in gastric cancer [51]. Inhibition of METTL3 enhances the sensitivity of pancreatic cancer cells to chemotherapy and radiotherapy [52]. High expression of METTL3 serves as an independent prognostic indicator in CC, and METTL3 presents oncogenic potencies in the development of CC [28,53,54]. In agreement with previous findings, we verified that METTL3 expression was elevated in CC samples and cells compared to that in normal tissues and cells. Upregulation of METTL3 determines poor prognosis in CC patients. Our results confirmed that the knockdown of METTL3 restrained the growth of CC cells both *in vivo* and *in vitro*. Additionally, silencing METTL3 suppressed CC cell migration.

ACIN1, also known as acinus, is fundamental for chromatin condensation during apoptosis [55]. In addition, ACIN1 is directly implicated in RNA processing, including transcription, mRNA splicing, and metabolism [56]. Emerging evidence indicates that ACIN1 is abundantly expressed in lung cancer patients compared to that in healthy individuals and that a high methylation level of ACIN1 predicts a high risk of lung cancer [57,58]. Using LinkedOmics and correlation analysis, we uncovered a strong and positive correlation between ACIN1 and METTL3. Moreover, our findings revealed that depletion of METTL3 inhibited m6A methylation of ACIN1. The results of rescue experiments supported the inference that METTL3 triggered the malignant behavior of CC cells through the regulation of ACIN1.

IGF2BP3 is a member of a novel family of m6A readers that are involved in modulating mRNA stability [59]. These findings demonstrate that IGF2BP3 plays an oncogenic role in the initiation and progression of CC by affecting mRNA stability and translation of target genes in an m6A-mediated manner [39,60,61]. Considering our data that METTL3 promotes the stabilization of ACIN1 mRNA to enhance its expression, we investigated the participation of IGF2BP3 in this pathway. The recovery of ACIN1 mRNA and protein levels in METTL3-downregulated CC cells was attributed to IGF2BP3 overexpression. In addition, it was confirmed that the enforced expression of IGF2BP3 alleviated the decay rate of ACIN1 mRNA in response to actinomycin D. Consistent with previous reports, we further confirmed that IGF2BP3 was responsible for the effects of METTL3 on the mRNA stability of ACIN1.

## Conclusion

This study elucidated the function and mode of action of METTL3 in CC pathogenesis. Our study illustrated that elevated METTL3 levels were associated with poor prognosis in of CC patients. Notably, the silencing of METTL3 retarded CC cell growth and migration by weakening ACIN1 mRNA stability via an m6A-IGF2BP3-dependent mechanism, expanding our in-depth knowledge of METTL3 in CC progression and unraveling the possibility of using METTL3 as a therapeutic target for CC therapy.
